# Multilayered Thin Films from Boronic Acid-Functional Poly(amido amine)s As Drug-Releasing Surfaces

**DOI:** 10.1007/s11095-015-1734-y

**Published:** 2015-06-26

**Authors:** Sry D. Hujaya, Johan F. J. Engbersen, Jos M. J. Paulusse

**Affiliations:** Department of Controlled Drug Delivery, MIRA Institute for Biomedical Technology and Technical Medicine, Faculty of Science and Technology, University of Twente, P.O. Box 217, 7500 AE Enschede, The Netherlands

**Keywords:** biodegradable polymers, chondroitin sulfate, dynamic covalent chemistry, layer-by-layer assembly, poly(vinyl alcohol)

## Abstract

**Purpose:**

To evaluate the potential of poly(amido amine)-based multilayered thin films in surface mediated drug release.

**Methods:**

Multilayered thin films were prepared from copolymers of phenylboronic acid-functional poly(amido amine)s and chondroitin sulfate (ChS) in the presence of Alizarin Red S (ARS) as a reporter molecule. Multilayer buildup and ARS incorporation were evaluated with UV–vis spectroscopy. Glucose responsiveness of the multilayers was investigated. Finally, cellular uptake of ARS by COS-7 cells grown on the films was assessed.

**Results:**

Multilayers based on alcohol containing polymers (ABOL-BA-PAA#ChS + ARS) displayed higher ARS incorporation than multilayers based on amine-containing polymers (DAB-BA-PAA#ChS + ARS). At physiological pH, a swift initial release of up to ~40% of the ARS content was observed during the first 12 h of incubation, followed by a much slower, gradual release of ARS. The multilayers were further evaluated by culturing COS-7 cells on top of multilayer-coated well plates. Cellular uptake of the fluorescent ARS-boronate ester was quantified through flow cytometry, and a maximum uptake of up to 30% was observed. Confocal microscopy confirmed the presence of ARS-boronate ester-containing particles in the nuclei of cells.

**Conclusions:**

The investigated multilayered thin films are effective in surface-mediated delivery of the model compound ARS. These multilayered surfaces are promising as drug-releasing delivery surface for coating stents, prostheses, and other implants.

**Graphical Abstract:**
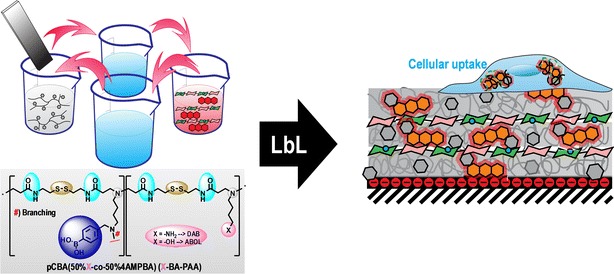
ᅟ

## Introduction

The coverage of the surface of materials for biomedical applications with multilayered thin films offers the possibility of providing to these materials a delivery system that is controllable in both the amount of loading, as well as the release of bioactive compounds (1,2). The amount of incorporated drug can be tuned by the number of layers in the film, which may reduce side effects and wasting of expensive drugs. Release profile and responsiveness can be altered by careful choice of appropriate macromolecules as main multilayer components (3). Moreover, highly localized delivery of incorporated drugs can be achieved, which may be beneficial for applications such as providing anti-inflammatory or anti-thrombotic properties to the surface of an implant.

Unlike hydrogel systems, where small molecules can be easily incorporated by simply dissolving the compound of interest in the hydrogel-forming solutions, incorporating small molecules via the layer-by-layer (LbL) alternate dipping technique requires that the molecule has strong enough association to at least one of the macromolecules forming the layers to shift the equilibrium away from the entropically more stable dissociated state and to prevent rapid diffusive release from the films. This requirement cannot be easily met, and is particularly difficult to achieve by electrostatic interactions alone. Therefore, several techniques are commonly utilized, as reviewed by Pavlukhina and Sukhishvili (4), such as using drug crystals as the substrate for multilayer build-up (5,6), interlayer crosslinking (7), pre-encapsulation of the drug molecules into micelles or particles (8), linking the drug molecules to one of the macromolecules either covalently (i.e. in a pro-drug approach) (9) or through the use of host-guest interactions (10), and other more specific complexation/interaction mechanisms (11).

An interesting alternative possibility for biologically active molecules possessing a vicinal diol functionality is the utilization of reversible binding of this diol group to boronic acids. The functional boronic acid (BA) moiety has received a lot of attention in the biomedical field, especially in the development of sensor, due to its ability to selectively form dynamic coordinative covalent esters with various diol-containing species such as glucose (12). In responsive drug delivery applications, the dynamic nature of boronate ester formation has been used to provide glucose responsiveness for treatment of diabetes, obesity, cancer and HIV (13), and to provide binding to the saccharides at the surface of mammalian cells (14,15). More interestingly, in hydrogel systems this dynamic covalent interaction provides the hydrogels with the ability to self-heal and adopt the specific shape of containers or cavities, a desirable property for wound-dressing and topical applications (16,17).

Parallel to the increasing interest in boronate ester formation, ARS has emerged as one of the most commonly used reporter molecules to analytically determine the binding constants of BA moieties to diols under relevant conditions (18,19). This practice is attributed to the particular fluorescence properties of ARS upon ester formation with a BA moiety under physiologically relevant conditions. Structurally, the anthraquinone derivative ARS contains a catechol moiety responsible for the strong binding with BA. ARS can therefore be seen as a model for catechol-containing drugs such as dopamine, epinephrine/adrenaline, and many others with main pharmacological applications in the nervous system (treatment of Parkinson’s, schizophrenia, attention deficit hyperactivity disorder (ADHD)), lung diseases (chronic obstructive pulmonary disease (COPD), asthma), and heart diseases (cardiac arrest, anaphylaxis, hypertension) (20). The characteristic fluorescence properties of the ARS-boronate ester may also assist in the characterization of loading, release, responsiveness and/or competitiveness with other diol species and even for additional imaging purposes (15).

Previously, we reported the preparation and properties of multilayered systems prepared from boronic acid-containing poly(amido amine)s (BA-PAA) and chondroitin sulfate (ChS) (21). Multilayer build-up of these films proceeds through electrostatic interactions, mainly through the positively-charged BA-PAA polymer chains and the negatively-charged sulfate-containing ChS. Though possible, boronate ester formation is not observed at a functional level for biomedical applications, i.e. the binding between the BA-moieties in the BA-PAA polymer and the diol groups in ChS is so weak that the BA-moieties are largely present in their boronic acid state. Here we describe our study into the use of BA-moieties in multilayered BA-PAA films for reversible drug binding and triggered release, using ARS as the model drug molecule.

## Materials and Methods

Chondroitin 4-sulfate sodium salt from bovine trachea (ChS, ≤10% water), alizarin red S (ARS), glucose (≥99.5%), glutathione (≥98.0%), sulfuric acid (H_2_SO_4_, 95–98%), and hydrogen peroxide (H_2_O_2_, 30 wt% in H_2_O) were purchased from Sigma-Aldrich (Zwijndrecht, The Netherlands). Sodium dihydrogen phosphate monohydrate (NaH_2_PO_4_.H_2_O, 99.0–102.0%), disodium hydrogen phosphate dihydrate (Na_2_HPO_4_.2H_2_O, 99.5%), citric acid (≥99%), and trisodium citrate dihydrate (≥99%) were purchased from Merck (Darmstadt, Germany). Solvents were of reagent grade and used without further purification unless otherwise noted. Milli-Q water (18.2 MΩ∙cm at 25°C) was obtained from a Synergy® water purification system (Millipore).

DAB-BA-PAA and ABOL-BA-PAA were synthesized as described previously (21).

PBS buffer was prepared by dissolving 1.54 g of Na_2_HPO_4_.2H_2_O, 0.3 g of NaH_2_PO_4_.H_2_O, and 8.2 g of NaCl into 1.0 L of Milli-Q water and adjusting the pH to 7.4.

Citrate buffered saline (CBS buffer) pH 4, 5, and 6 were prepared by dissolving citric acid, trisodium citrate dehydrate and NaCl in the appropriate amounts in Milli-Q and adjusting the pH with HCl or NaOH.

UV characterization of multilayers was performed in the dry state using a UV-2401 PC (Shimadzu, ‘s-Hertogenbosch, The Netherlands) UV spectrophotometer. Each film fabricated on UV-transparent 7.5 × 37 × 1 mm quartz glass (Ted Pella, Redding, USA) was measured in three different arbitrary positions. Absorbance scan was carried out in the 200–700 nm wavelength range. All data points were then corrected for baseline offset by subtracting the absorbance value at 400 nm from each data point. Relative absorbance values were obtained by normalizing each data point with the respective value at time 0.

Poly-D-lysine-coated 96 well plates (PDL-TCPS) for multilayer build-up for cell culture and transfection experiments were purchased from Greiner (Alphen aan den Rijn, The Netherlands).

COS-7 cells (European Collection of Animal Cell Cultures (ECACC) Catalogue No. 87021302) were grown in DMEM containing 4.5 g/L glucose and GlutaMAX^TM^ (Invitrogen, Breda, The Netherlands) supplemented with 2% (v/v) PennStrepp (Lonza, Breda, The Netherlands) and 10% (v/v) fetal bovine serum (Lonza, Breda, The Netherlands).

Fluorescence microscopy was performed at 4X, 10X, 20X, and/or 40X objectives using EVOS digital inverted microscope (EMS, Wageningen, The Netherlands).

Confocal microscopy was performed on an LSM 510 (Carl Zeiss, Sliedrecht, The Netherlands) using the Zen 2009 software.

Fluorescence-activated cell sorting (FACS) was carried out in a Becton-Dickinson FACSCalibur (Breda, The Netherlands).

### Multilayered Thin Film Construction and Build-Up Profiles

Fresh BA-PAA solutions were prepared shortly before the start of multilayer build-up from the solid materials, which had been re-lyophilized overnight to avoid weighing errors due to their hygroscopic properties. All BA-PAA solutions (2.0 mg/mL) were prepared in PBS buffer at pH 7.4 to avoid possible variations in pH.

Prior to the assembly, quartz substrates (7.5 × 32 mm) were etched for 30 min in piranha acid to activate the surface, rinsed with copious amounts of Milli-Q water, and dried under N_2_ stream. These substrates were then dipped into DAB-BA-PAA or ABOL-BA-PAA solution (2 mg/mL in PBS buffer pH 7.4) for 5 min, transferred into washing solution containing PBS buffer for 1 min, dipped briefly in a large amount of Milli-Q water, transferred into ChS + ARS (2 mg/mL ChS and 1 mM ARS in Milli-Q water) solution for 5 min, dipped into the second washing solution containing Milli-Q water for 1 min, and finally followed by another brief dipping in Milli-Q. This cycle was repeated to reach the desired number of bilayer. Drying under N_2_ stream was performed after every BA-PAA layer deposition, excluding the very first layer. The resulting ensemble is denoted by BA-PAA-(ChS + ARS#BA-PAA)_**n**_, where BA-PAA represents either DAB-BA-PAA or ABOL-BA-PAA and **n** represents the number of bilayer. The first BA-PAA layer is regarded as a precursor layer and therefore excluded from the bilayer number count. Typically, the ensemble consists of 5 or 10 bilayers with the poly(amido amine) polymer as the last layer. For every multilayered system, three samples were fabricated in parallel to give estimation for standard deviation. To study the build-up profiles, UV spectra were recorded after each drying step following BA-PAA layer formation throughout the 200–700 nm range. Every absorbance values were then corrected for baseline shift at 700 nm. Afterwards, the multilayers were dipped into ChS + ARS solution to continue multilayer build-up.

Multilayers for cell culture were fabricated directly in the wells of poly-D-lysine-coated 96-well plates (PDL-TCPS, Greiner) by alternatingly dispensing deposition (70 μL) and washing (2x 100 μL) solutions under sterile conditions inside the laminar flow hood (LFH). Deposition started with ChS + ARS (2 mg/mL ChS and 1 mM ARS in Milli-Q water, 30 min for the first layer, 5 min next) as the first layer to a total of 10 bilayers ending with the BA-PAA layer. No intermediate drying steps were applied. At the end of the fabrication process, the plates were left inside the LFH briefly to dry the films. Coated plates were kept at 4°C and used as soon as possible (typically overnight). These multilayered samples are designated as PDL-(ChS + ARS#BA-PAA)_**10**_ to indicate the presence of a PDL layer as a precursor layer. Compared to multilayers built on quartz and silicon wafer substrates, these systems substitute the first BA-PAA layer with PDL layer inherent to the well plate surface.

### ARS Release Under Physiological Conditions and At Acidic pH

ARS release profiles of the three multilayered systems in PBS buffer pH 7.4 at 37°C were investigated by dipping the thin films formed on quartz slides in 2 mL of PBS buffer pH 7.4 solution and incubating them in a water bath with temperature set to 37°C. At regular intervals, the samples were removed, briefly dipped in a large amount of Milli-Q water, dried under N_2_ stream and measured by UV–vis spectrophotometer. The release study at acidic pH (pH 4, 5, and 6) was carried out in a similar fashion, but using citrate buffered saline (CBS) instead of PBS.

### ARS Release Under Various Reductive Conditions

Degradability of the multilayered systems was investigated in a similar way as for the investigation of their respective stability profiles under physiological conditions, but in the presence of 0.4 mM or 10 mM glutathione in the incubation medium. Solutions containing glutathione in PBS buffer at pH 7.4 were prepared fresh directly prior to the start of experiment. Due to instability of glutathione in PBS buffer at pH 7.4, no solution of more than three hours old was used.

### ARS Release in the Presence of Glucose

The influence of glucose concentration on ARS release profile was investigated in a similar way as for the investigation of their respective stability profiles under physiological conditions, but in the presence of 25 mM and 100 mM of glucose. The 25 mM concentration is used to mimic the glucose concentration in cell culture medium. This concentration is roughly 2.5 times the minimal blood glucose level in diabetic patients (22).

### Particle Uptake by COS-7 Cells: FACS

To investigate the possibility of COS-7 cells taking up ARS-boronate ester from the multilayer surface, cells were seeded directly on multilayer-coated wells at 20 000 cells/well or 62 500 cells/cm^2^ in complete medium with serum and left to proliferate at 37°C under humidified atmosphere with 5% CO_2_.

After 0, 3, 6, 12, 24, and 48 h, cells were trypsinized, centrifuged (5 min, 600 g), and analyzed by FACS. Cells were also cultured for 12 h on regular polystyrene (PS) culture plates with and without the addition of free ARS and on multilayered films without ARS as controls to determine live cell population and fluorescence-positive cell population markers. On some multilayered samples treated similarly but without addition of cells, physical force was applied to break down the films into the cell culture medium. The resulting suspension was also analyzed through FACS to locate multilayer remnant population and identify their relative fluorescence intensity.

Excitation of ARS-boronate ester was performed at 488 nm and emission was detected via a 585 nm band-pass filter. At least 20 000 – 30 000 total events were measured to reach 10 000 events for gated living cells. Data analysis was performed using the FACS Cellquest Software. The gate setting was equal for all samples within the same experiment. Dot plots were applied to separate living cells population from dead cells and film residues and particles. From the histogram obtained, markers were drawn to identify cells as live positive cells or live negative cells.

### Particle Uptake by COS-7 Cells: Confocal Microscopy

Samples for confocal microscopy were prepared by culturing cells as described in previously. Following 24 h of culture, cells were trypsinized and recultured inside a cover glass slide imaging chamber (General Electric Healthcare, Eindhoven, The Netherlands) for 6 h to allow the cells to attach. The cells were then washed with PBS, fixed with 3.7% paraformaldehyde for 15 min at RT, washed 3 times with PBS, and stained with Hoechst 33258 (2 μL/mL in PBS) for 15 min at RT. Samples were then washed 3 times with PBS and mounted on a clean microscope glass slide using aqueous mounting medium (Ibidi, Munich, Germany) and sealed with nail polish.

Confocal microscopy was performed on an LSM 510 (Carl Zeiss, Sliedrecht, The Netherlands) using Zen 2009 software. Both fluorophores (BA-ARS and Hoechst 33258) were excited using a 543 nm argon laser passed through HFT KP 700/S43 and split through an NFT 490. BA-ARS fluorescence was analyzed past a BP 565–615 IR filter and Hoechst 33258 excitation was analyzed past a BP 390–465 IR filter. Z-stack sectioning was performed over 20 slices throughout the 33.75 μm height range.

## Results and Discussion

### Multilayered Thin Film Build-Up

**Scheme 1:**
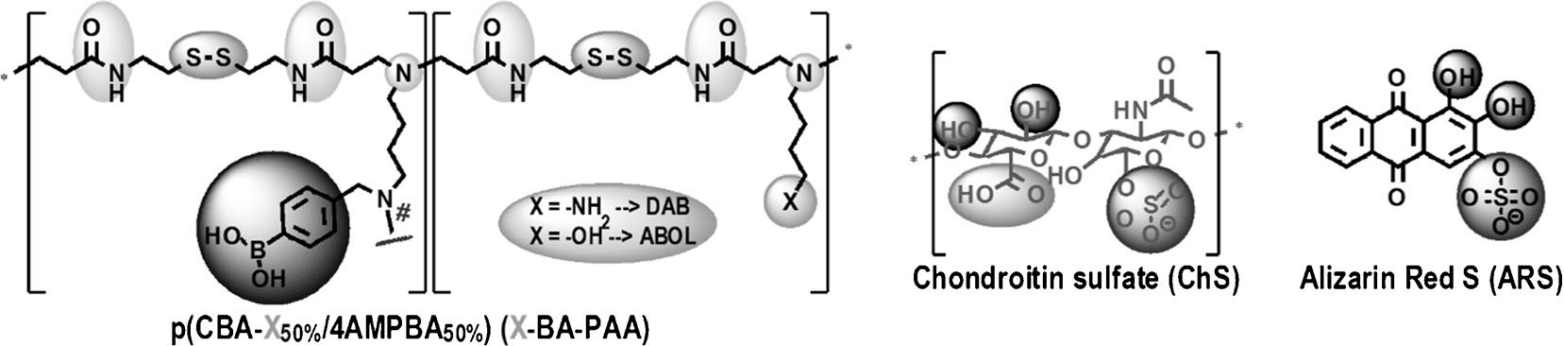
Chemical structures of DAB-BA-PAA, ABOL-BA-PAA, ChS, and ARS.

We previously reported the syntheses of two branched boronic acid-functionalized poly(amido amine)s (BA-PAAs) (21). The degrees of BA functionalization of the two BA-PAAs were similar at ~50%, with branching estimated to be ~10 and ~30% for DAB-BA-PAA and ABOL-BA-PAA, respectively, and number average molecular weights of 12 and 14 kg/mol, respectively. Chemical structures of the two polymers, ChS, and ARS utilized in this study are presented in Scheme 1. These polymers were employed in multilayer build-up as described in the Materials and Methods section.

**Fig. 1:**
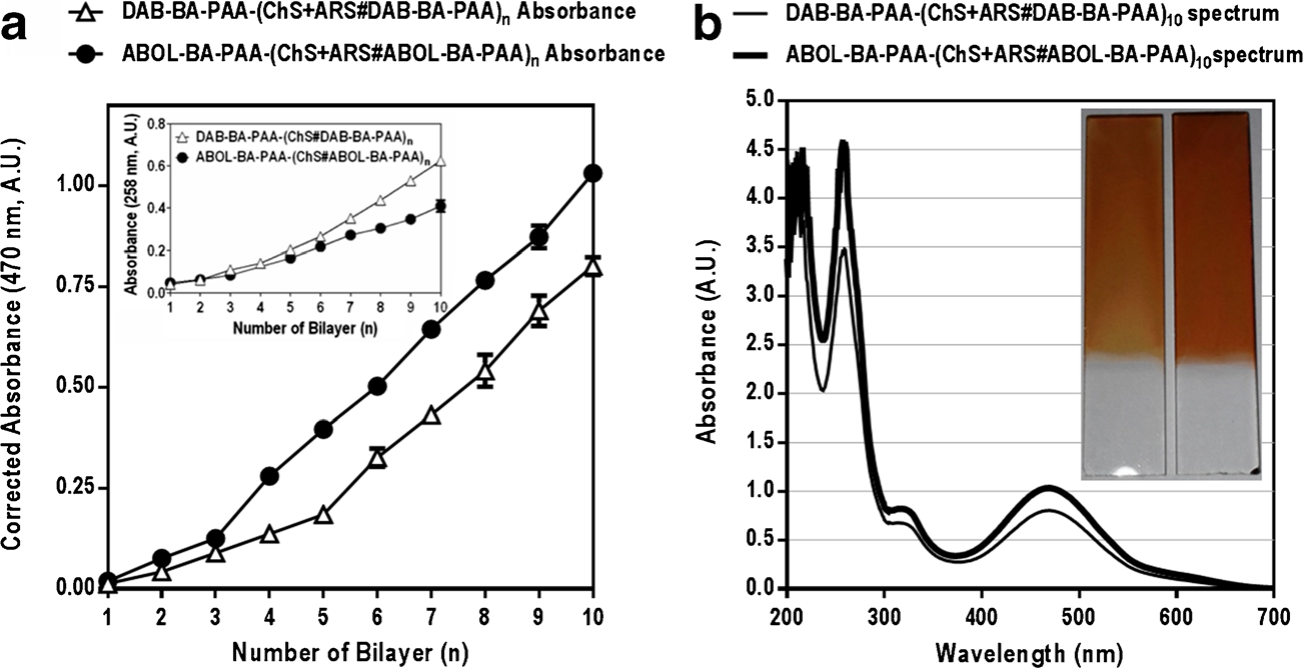
(**a**) UV-Absorption-based build-up profiles (470 nm) and (**b**) UV spectra of DAB-BA-PAA-(ChS + ARS#DAB-BA-PAA)_n_ and ABOL-BA-PAA-(ChS + ARS#ABOL-BA-PAA)_n_ in the dry film. Inset in (**a**) shows the build-up profiles of multilayers in the absence of ARS; inset in (**b**) shows digital photograph of DAB-BA-PAA-(ChS + ARS#DAB-BA-PAA)_10_ (*left*) and ABOL-BA-PAA-(ChS + ARS#ABOL-BA-PAA)_10_ (*right*).

Figure 1 shows build-up profiles of DAB-BA-PAA-(ChS + ARS#DAB-BA-PAA)_**10**_ and ABOL-BA-PAA-(ChS + ARS#ABOL-BA-PAA)_**10**_ based on increases in absorbance at 470 nm. Incorporation of the dark red ARS drastically changes the spectral properties of the films, which complicates relating the UV spectra of these films with previous ARS-free multilayers (21). As the ARS is the reporter molecule for the investigation of both loading and release, and no multilayer build-up was observed in the absence of ChS, it was deemed appropriate to study the film behavior based on the UV-absorption of ARS. The opaque nature of the films is very reminiscent to the ARS-free system, indicating that ChS still makes up the majority of the material in the multilayered system, providing thick films.

Figure 1 indicates relatively linear build-up profiles for the two BA-PAAs. Both systems display a slow build-up in the early stages of deposition, probably due to incomplete substrate surface coverage, causing repulsion between the substrate and the negatively-charged ARS molecules. The subsequent linear increase of ARS absorbance values indicates that ARS is incorporated systematically as the bilayer number increases. Incorporation of ARS proved to be more efficient in the ABOL-BA-PAA multilayers than in the DAB-BA-PAA multilayers. This is in contrast to the previous study showing that DAB-BA-PAA facilitated deposition of ChS and resulted in overall thicker films, as compared to ABOL-BA-PAA (21). However, this study was based on the absorbance specific to ChS (at 258 nm) and therefore did not provide information on the amount of incorporated BA-PAA polymer. The higher ARS uptake in the ABOL-BA-PAA#ChS multilayers may indicate that ABOL-BA-PAA multilayers contain a higher ABOL-BA-PAA/ChS ratio than DAB-BA-PAA/ChS ratio of DAB-BA-PAA#ChS multilayers, thereby making it possible to incorporate more ARS into the films through BA-ARS interactions. At 5 and 10 bl, ABOL-BA-PAA-based films contain 2 and 1.3 times more ARS, respectively, as compared to DAB-BA-PAA-based films at the same bl number. The inset in the UV spectra of Fig. 1 shows the digital photograph of DAB-BA-PAA-(ChS + ARS#DAB-BA-PAA)_**10**_ and ABOL-BA-PAA-(ChS + ARS#ABOL-BA-PAA)_**10**_. The image reveals that DAB-BA-PAA-(ChS + ARS#DAB-BA-PAA)_**10**_ contains less ARS than ABOL-BA-PAA-(ChS + ARS#ABOL-BA-PAA)_**10**_.

With DAB-BA-PAA possessing higher positive charge density due to the protonated amines in the side chains, it may be expected that additional electrostatic interactions with the negatively charged ARS may help to incorporate additional ARS in the DAB-BA-PAA#ChS multilayer films. However, our previous attempts to incorporate various other charged small dye molecules through loading into multilayers purely by electrostatic interactions were unsuccessful. Often the dyes acted as salts, enhancing deposition of polyelectrolytes without being incorporated (unpublished results). It is therefore expected that ARS-boronate ester formation is the major driving force for the successful incorporation of ARS into the (BA-PAA#ChS) multilayers.

As a pH indicator, ARS with a pK_a_ of ~4.5 is known to change color from yellow to red in the pH range of 3.5 – 6.5 (23). Throughout the multilayer build-up, ChS + ARS deposition solution in water was red in color (pH ~7), while the pH of the BA-PAA deposition solutions was maintained at 7.4 through the use of phosphate buffer. If the color of ARS in the constructs was determined solely by the pH of the deposition solutions, a red multilayered film with λ_max_ above 500 nm would be formed. However, the color of the films (Fig. 1, inset) was orange rather than red, with a λ_max_ at 470 nm. This λ_max_ of the multilayers at 470 nm was maintained throughout the 10 cycles of deposition investigated. It is known from the literature that the ARS-phenylboronate ester (BA-ARS) possesses a relatively high association constant (1300 M^−1^ at pH 7.4, 0.10 M phosphate buffer) (12). In contrast, ChS which contains vicinal diol groups with larger dihedral angles only forms labile boronate esters with BA, which are further weakened by electrostatic repulsion of the boronate anion by the sulfate and carboxylic acid groups of ChS. Therefore, the orange color is most likely attributed to boronate ester formation of ARS with phenylboronic acid (BA) moieties in the BA-PAA (18). This finding of multilayer λ_max_ at 470 nm is in agreement with previous findings by Ding *et al*. (24).

As further comparison, DAB-BA-PAA-(PVA + ARS#DAB-BA-PAA)_**10**_ – an ARS-loaded multilayer prepared with PVA instead of ChS – remained red instead of orange (data not shown), indicating significant competition between diols of PVA and ARS for ester formation with BA. Due to the multivalency and high local concentration of vicinal diol groups in PVA, the majority of BA moieties is bound to PVA, leaving the ARS molecules only physically entrapped or loosely bound in this multilayer.

It is important to note that the BA-PAA#ChS multilayer films have an opaque appearance, which especially manifests at higher number of layers. This hampers accurate UV determinations. Therefore, in order to minimize possible errors in UV characterization, the number of bilayers in the subsequent studies on pH-, glucose-responsiveness, and degradation of the layers has been limited to 5 bilayers.

### ARS Release Profiles Under Physiological Conditions and At Acidic pH

**Fig. 2:**
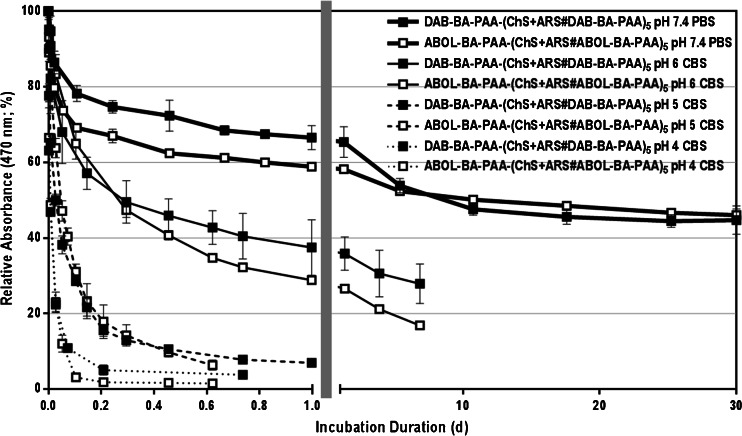
ARS release profiles under physiological conditions and acidic pH *in vitro*. Left part of the abscissa shows release within 1 day; right part shows slower release during remaining incubation.

Due to the pH-sensitive nature of the boronate ester formation (12), ARS is expected to be rapidly released at acidic pH where boronate ester formation is less favorable. To investigate the ARS release profiles from the multilayered systems under physiological conditions and at acidic pH, the films were incubated in PBS at pH 7.4, and in CBS at pH 6, 5, or 4 at 37°C. The ARS release profiles are depicted in Fig. 2 indicating that within 12 h of incubation under physiological conditions (pH 7.4), about 30% and 40% of the incorporated ARS was released for DAB-BA-PAA and ABOL-BA-PAA systems, respectively. ARS release continued, though substantially slower, reaching a plateau after 10 days of incubation where ~50% of the incorporated ARS was released from the multilayered constructs. The rapid release of ARS under physiological conditions may be facilitated by entropic gain from the liberation of ARS molecules before finally reaching an equilibrium at ~50% ARS content.

ABOL-BA-PAA multilayers, possessing a higher loading of ARS than the DAB-BA-PAA multilayers, released more of the ARS content during the initial rapid release phase. This means that under equal conditions and at the same number of bilayers, much more ARS is released to the incubation medium by the ABOL-BA-PAA system, than in the case of the DAB-BA-PAA system. Also, since the release was only followed by the decrease in ARS absorbance in the multilayers, it is not deducible from these results whether or not also BA-PAA polymers or ChS were released during the initial release phase. Our previous results with ARS-free multilayers showed that up to ~20% of the polymeric components of the multilayers were also released within 2 h of incubation under the same conditions (21). Therefore, the release observed in Fig. 2 may represent the release of a mixture of both free ARS and ARS-boronate ester complexes.

At acidic pH, ARS-boronate ester is less stable (12) and leads to more rapid ARS release. At pH 6, at least two times faster release of ARS is observed, as compared to the release at pH 7.4. At more acidic pH the release proceeds progressively faster, and complete ARS release was achieved at pH 5 and 4, after 17 h and 5 h, respectively. In relation to the potential application where these multilayers are used to coat the surface of implantable biomaterials or devices, low-pH-triggered release may be exploited to deliver diol- or catechol-containing anti-inflammatory drugs (25,26). Since implantation surgery is followed by inflammatory response, decreasing pH locally, these multilayered coatings may help in reducing inflammation by delivering the correct dosage based on the change in pH of the environment.

**Scheme 2:**
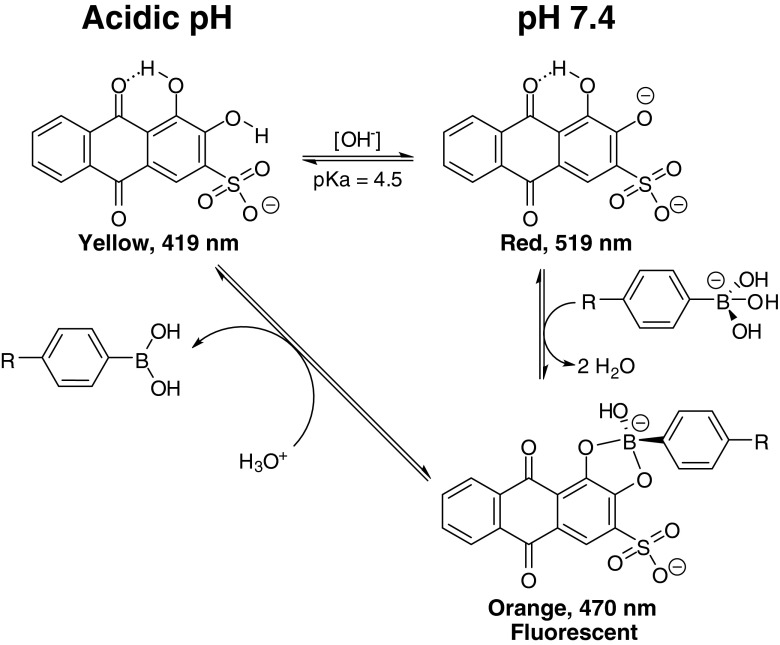
Equilibria of various ARS species at acidic and neutral pH, and respective boronate ester formation with BA moieties.

**Fig. 3:**
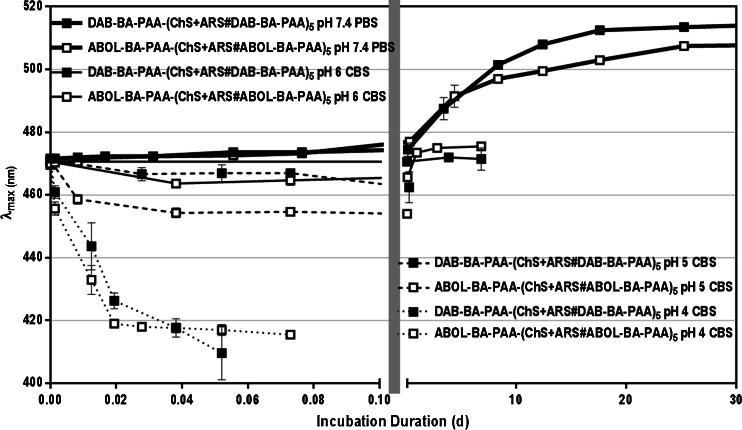
Maximum wavelength shift of ARS molecules within DAB-BA-PAA-(ChS + ARS#DAB-BA-PAA)_5_ and ABOL-BA-PAA-(ChS + ARS#ABOL-BA-PAA)_5_ films at various pH.

As discussed previously, ARS is a pH indicator. The equilibria between the different ARS species are depicted in Scheme 2, including those in the presence of BA moieties. In the presence of these moieties, the high binding constant between ARS and BA drives the equilibrium towards boronate ester formation with a distinctly different λ_max_. This boronate ester is only stable at neutral to basic pH, where it is predominantly present in the negatively charged tetrahedral form (12). Therefore, upon decrease of pH, the equilibria are shifted towards free ARS, which are then shifted further to the yellow species (λ_max_ = 419 nm) by the low pH. Figure 3 shows the expected decrease in λ_max_ during the incubation in the different acidic pH values. As shown in Scheme 2, this shift in λ_max_ is accompanied by ARS-boronate ester hydrolysis, leading to ARS release from the films.

During the first hour of exposure to PBS buffer at pH 7.4, the λ_max_ of the multilayers remains stable at 470 nm, followed by a shift to longer wavelength throughout the rest of the incubation period. The shift in λ_max_ reached a maximum value at ~510 nm after about 20 days of incubation. Unlike in the case of acidic pH, this shift in λ_max_ progressed much more slowly, underlining the high binding affinity of the BA to ARS at pH 7.4. The initial 1 h rapid release of ARS (Fig. 2) is not accompanied by a shift in λ_max_. This further indicates most of the incorporated ARS is bound to BA moieties or involved in dynamic ester formation (27) with the BA moieties in the film. In the later stages of incubation, slow gradual boronate ester hydrolysis and diffusion of ARS take place, leaving higher portions of unbound physically-entrapped ARS in the multilayer, resulting in a shift of λ_max_ to that corresponding to free ARS at pH 7.4 (λ_max_ = 519 nm).

### ARS Release Under Reducing Conditions

**Fig. 4:**
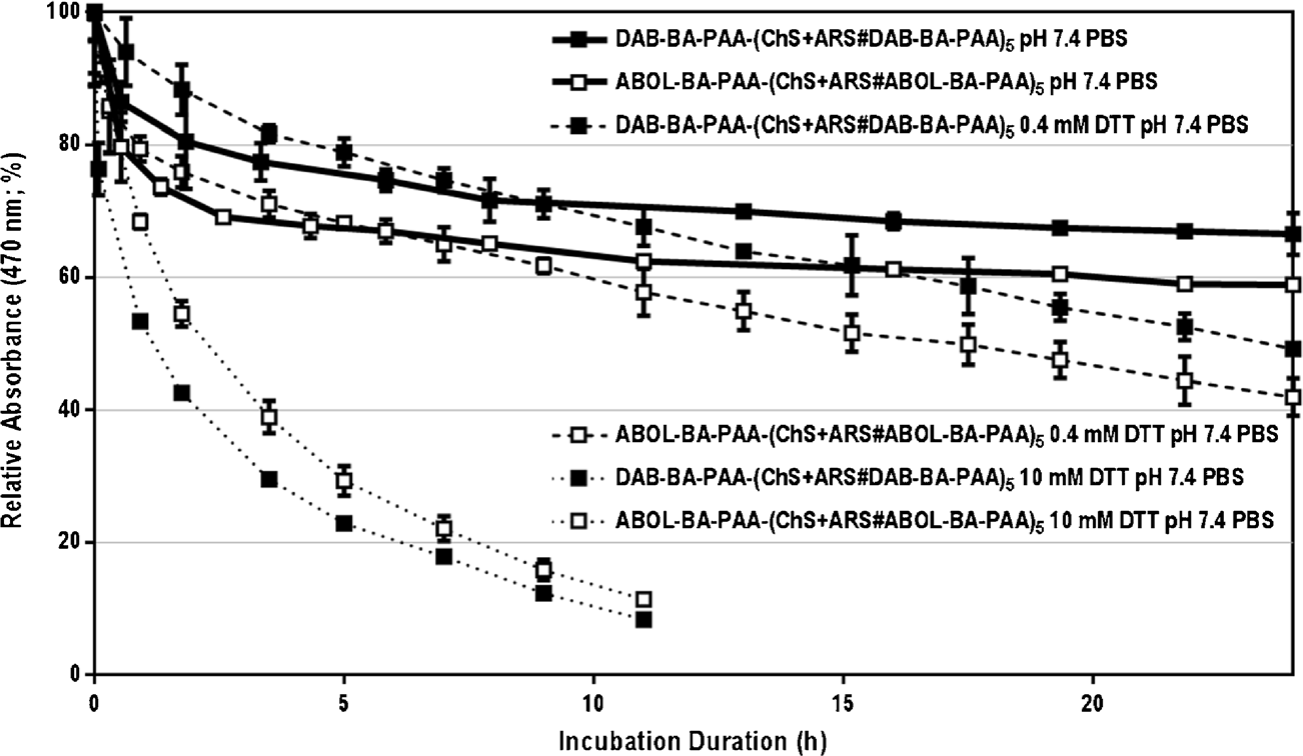
Degradation profiles of DAB-BA-PAA-(ChS + ARS#DAB-BA-PAA)_5_ and ABOL-BA-PAA-(ChS + ARS#ABOL-BA-PAA)_5_ in PBS at pH 7.4 containing 0.4 or 10 mM glutathione at 37°C *in vitro*.

Owing to the presence of disulfide bonds in the main polymer chain of the BA-PAAs, the resulting multilayered ensembles are responsive to the presence of reducing agents as well. This reducibility is demonstrated by exposing the multilayers to 0.4 mM and 10 mM of glutathione in the incubation solutions of PBS at pH 7.4. Figure 4 shows the ARS release profiles under these reducing conditions. In the presence of 0.4 mM glutathione, ARS release is not significantly enhanced as compared to that observed in the absence of glutathione, at least during the first 10 h of incubation. This slow response to 0.4 mM glutathione was also observed for the degradation of the ARS-free multilayers, and can be attributed to their relatively high structural stability due to the branched structure of the polymers. However, during prolonged incubation, disulfide reduction progressed more extensively (incubation duration > 10 h), ARS release displayed an increased release compared to the release observed in the absence of glutathione. At the higher glutathione concentration of 10 mM the accelerated release was more pronounced and complete ARS release and film degradation was achieved after 10 h of incubation.

#### ARS Release In The Presence of Glucose

**Fig. 5:**
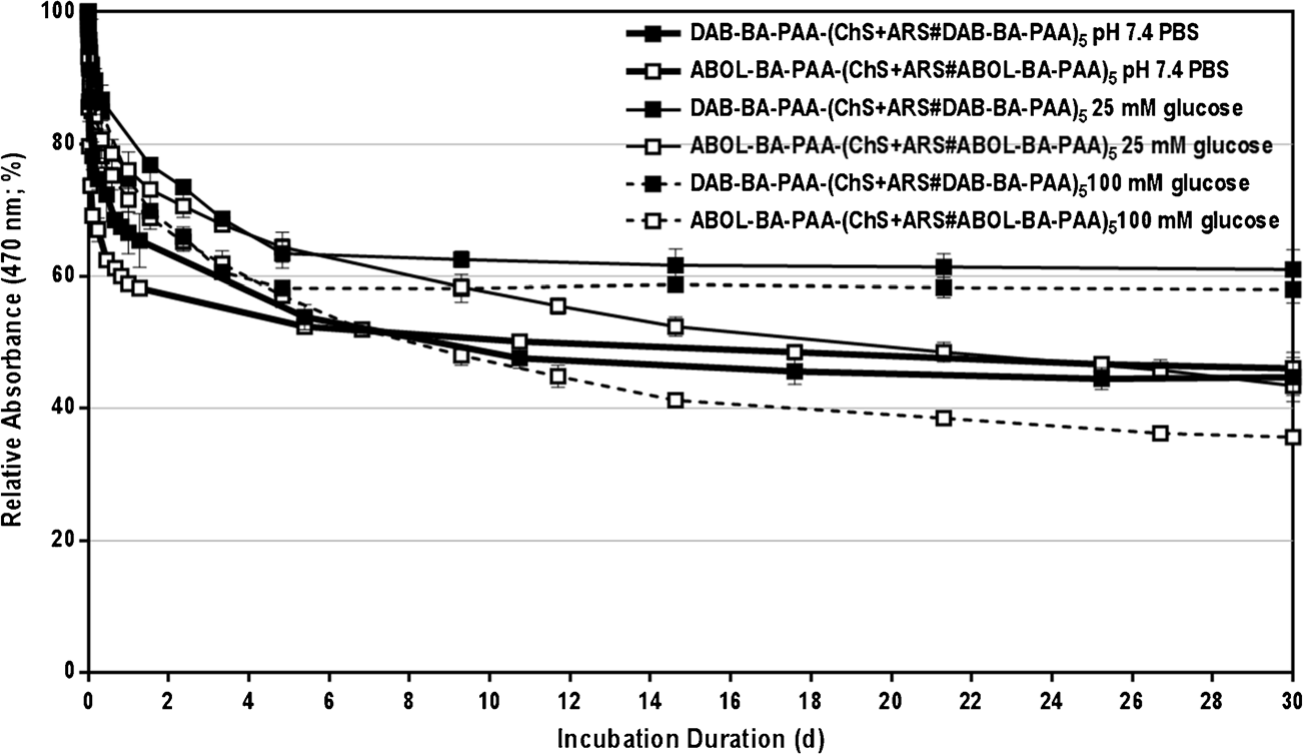
ARS release profiles under physiological conditions and in the presence of 25 and 100 mM glucose *in vitro*.

As a diol-containing molecule, glucose may compete with ARS in binding to the BA-moieties in the (BA-PAA#ChS) multilayers, which can result in a glucose-triggered ARS release from the multilayers. However, the results in Fig. 5 show that ARS release is only slightly increased by the presence of glucose at elevated concentration of 100 mM, and even decreased at 25 mM glucose concentration. At 25 mM glucose concentration, the slightly slower initial ARS release, as compared to the release in the absence of glucose, is probably due to increases in osmotic pressure in the incubation medium, leading to an overall effect of decreased ARS diffusion from the films. In general, the absence of any significant glucose-response is likely due to the much stronger binding constant of BA with ARS than with glucose (i.e. ~300-fold stronger (12)). Furthermore, pronounced ARS release may have been further hampered by slow diffusion of glucose into and ARS out of the ChS-rich multilayered construct.

Figure 5 further shows that albeit minor, ABOL-BA-PAA (white symbols) displayed slightly more increased responsiveness to glucose than DAB-BA-PAA (black symbols). At the same glucose concentration of either 25 or 100 mM, more pronounced ARS release was observed for ABOL-BA-PAA multilayers than for DAB-BA-PAA systems. This may be attributed to the weaker interaction between the neutral primary alcohol side groups of ABOL-BA-PAA with the negatively-charged ChS, as compared to the more positively-charged primary amines of DAB-BA-PAA. In the former case, weaker interaction may lead to pronounced swelling that enhances ARS release, while in the latter case, presence of additional positive charges may restrict release due to electrostatic interaction between ARS and the protonated amines.

**Fig. 6:**
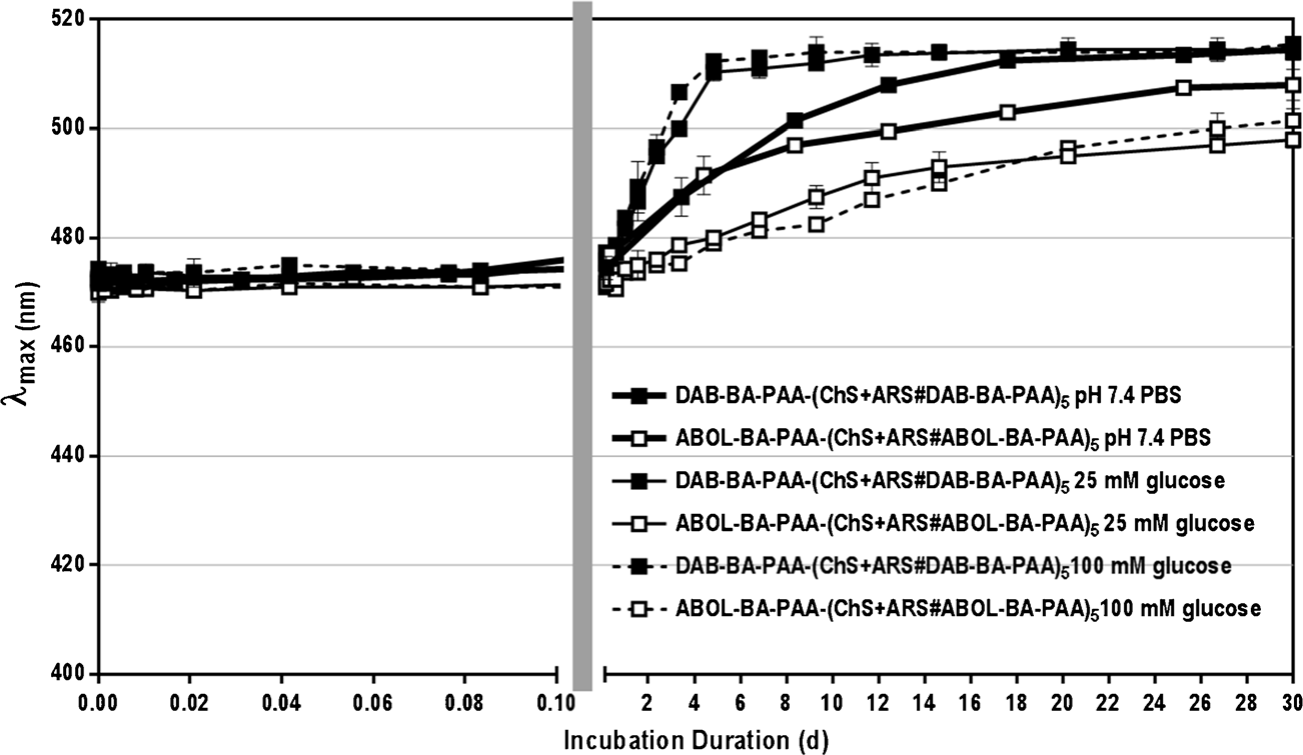
Maximum wavelength shift of ARS molecules within DAB-BA-PAA-(ChS + ARS#DAB-BA-PAA)_5_ and ABOL-BA-PAA-(ChS + ARS#ABOL-BA-PAA)_5_ films in the presence of 25 and 100 mM glucose.

The change in λ_max_ throughout the incubation in glucose solution (Fig. 6) shows a distinct trend. The λ_max_ of DAB-BA-PAA multilayers displays a more rapid red-shift (pointing to free ARS) than ABOL-BA-PAA, with no significant dependence on glucose concentration. A similar trend was observed for films incubated under physiological conditions. DAB-BA-PAA multilayers eventually have higher λ_max_ than ABOL-BA-PAA multilayers, which could indicate that the ARS-boronate ester in DAB-BA-PAA multilayers is weaker than in the ABOL-BA-PAA system. The pronounced red-shift of λ_max_ is probably enhanced by the lower diffusion coefficient of negatively-charged ARS from the multilayers, which contains higher amounts of positively-charged primary amine side groups. Thus, it is likely that in a typical DAB-BA-PAA multilayer, ARS is more readily freed from the boronate ester with BA, but remains physically entrapped within the multilayers, hence resulting in a change in λ_max_.

**Scheme 3:**
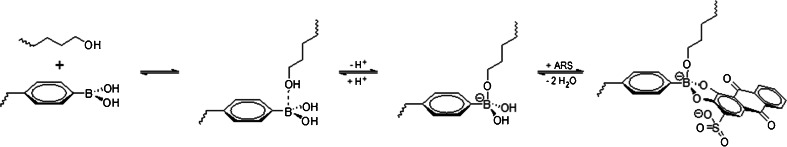
Acid–base equilibria between trigonal boronic acid and vicinity primary alcohol side group of ABOL-BA-PAA and the subsequent boronate ester formation with ARS.

The increased stability of ABOL-BA-PAA with ARS as compared to DAB-BA-PAA, may be the primary alcohol side groups of ABOL-BA-PAA in the proximity of BA, which may provide additional stabilization of the ARS-boronate ester. It is known that boronate esters are more stable in their tetragonal anion form (12); in a compact layer of BA-PAA, primary alcohols in the vicinity may donate their free electron pairs, effectively lowering the apparent pKa of the BA (Scheme 3). In contrast, as the majority of primary amines in DAB-BA-PAA is protonated, less electron pair donation can be provided by these groups.

### Uptake of ARS by COS-7 Cells Cultured on Multilayers

In this study, multilayered thin films of (ChS + ARS#BA-PAA) are investigated as a drug delivery system with ARS as a reporter molecule. As observed through the previous release study, these multilayers may serve as a drug reservoir to provide sustained release under physiological conditions or triggered release by changes in pH or through reducing conditions.

The ARS-boronate ester within the multilayered surface is fluorescent and may serve as a fluorescent label for the detection of cellular uptake of ARS-boronate esters. These ARS-boronate esters may be released from the multilayers either in the form of nanoparticles in which ARS is embedded and covalently bound to BA as boronate ester, or as more labile BA-PAA aggregates with weaker ARS binding. Earlier it has been found that ABOL-BA-PAA polymers can self-assemble into nano-sized particles (15). Dynamic light scattering studies on 0.6 mg/mL solutions of ABOL-BA-PAA in 50 mM HEPES buffer at pH 7.4 revealed the presence of homogeneous particles of ~94 nm, with a positive zeta potential (up to +40 mV). The same solution of DAB-BA-PAA showed higher polydispersity and lower zeta potential (+4 mV) (data not shown). These observations may be an indication that multilayers of ABOL-BA-PAA may provide a larger amount of ARS-complexed nanoparticles than the DAB-BA-PAA multilayers. Such an effect could be relevant for cellular uptake of ARS by cells cultured on top of the multilayers, since nanoparticles with positive zeta potential are more readily internalized by cells and contain more stable and longer lasting BA-ARS complexes.

To investigate whether ARS can be delivered by the films to become internalized by cells cultured on the multilayered surfaces, COS-7 cells were cultured on 96-well poly-D-lysine-coated tissue culture-treated polystyrene (PDL-TCPS), coated with (ChS + ARS#BA-PAA)_**10**_. The degree of cellular uptake of polymer-bound ARS was determined at 0, 3, 6, 12, 24, and 48 h time points after seeding of the cells on the multilayers. After the designated culture duration, cells were trypsinized, fixed and stored at 4°C before being measured at the end of the experiment. Trypsinization was found to not cause dissolution of the multilayered surface (data not shown). In addition to the cells cultured on the (ChS + ARS#BA-DAB)_**10**_ (entry #1) and (ChS + ARS#BA-ABOL)_**10**_ (entry #2) multilayers, several controls were prepared for a more thorough analysis as elaborated in Table I. Multilayered systems without cells (entries #5–8) served as a guideline to separate live cell populations from the population of film debris and/or particles in the FACS analysis. Cells seeded on PS well plates without multilayers (entry #9) served as negative control to help gate for live cells. Cells seeded on multilayers without ARS, (ChS#DAB-BA-PAA)_**10**_ (entry #3), and (ChS#ABOL-BA-PAA)_**10**_ (entry #4), and on PS with additional ARS in the medium (entry #10) served as a guideline to establish a marker to separate positive cells (i.e. cells that have taken up fluorescent ARS-boronate ester) from negative cells, and to verify that detected fluorescence is only from the ARS-boronate ester. The degree of cellular uptake is presented in two ways; i.e. 1) as percentage of positive cells in the live cell population, and 2) as relative fluorescence intensity of sample suspensions.

**Table I:** List of Samples and Controls Incorporated During Cellular Uptake Studies and Their Respective Abbreviations

Entry	Sample name	Category	Abbreviation
1	(ChS + ARS#DAB-BA-PAA)_**10**_ with cells	ARS	DAB-BA-PAA
2	(ChS + ARS#ABOL-BA-PAA)_**10**_ with cells		ABOL-BA-PAA
3	(ChS#DAB-BA-PAA)_**10**_ with cells	No ARS	DAB-BA-PAA no-ARS
4	(ChS#ABOL-BA-PAA)_**10**_ with cells		ABOL-BA-PAA no-ARS
	Controls without cells		
5	(ChS + ARS#DAB-BA-PAA)_**10**_ without cells	No cells	DAB-BA-PAA no-cell
6	(ChS + ARS#ABOL-BA-PAA)_**10**_ without cells		ABOL-BA-PAA no-cell
7	(ChS#DAB-BA-PAA)_**10**_ without cells	No ARS, no cells	DAB-BA-PAA no-ARS-no-cell
8	(ChS#ABOL-BA-PAA)_**10**_ without cells		ABOL-BA-PAA no-ARS-no-cell
	Controls without multilayer		
9	PS with cells	PS	PS
10	PS with cells seeded in medium containing ARS	PS + ARS	PS + ARS

**Fig. 7:**
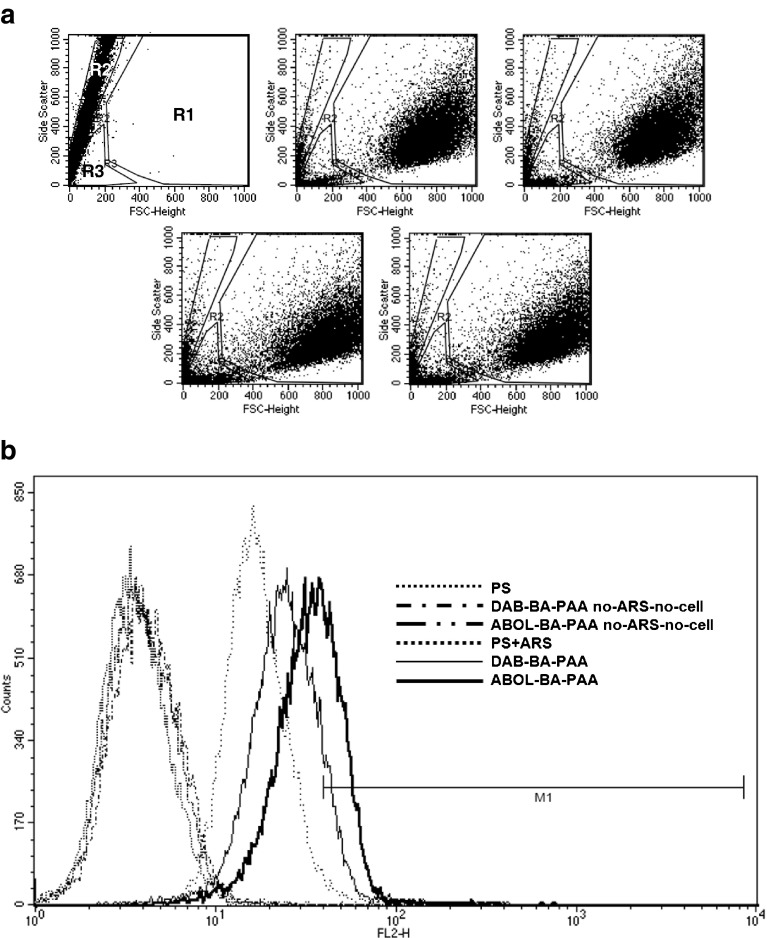
(**a**) Representative dot plots of: (from *left* to *right*, *top* to *bottom*) multilayer fragments (see text), PS, PS + ARS, DAB-BA-PAA, and ABOL-BA-PAA after 12 h of culture duration. Three regions are selected on every dot plot, namely R1 for live cells (largest region to the *right*), R3 for dead cells (*bottom left corner*), and R2 (*left part of dot plot*) most apparent for multilayer fragment populations shown in the first dot plot. (**b**) Overlay histogram of samples gated for live cells (G1 = R1).

Typical dot plots for the different conditions are shown in Fig. 7a. From the different plots, three regions can be distinguished representing populations of live cells (R1), particles or multilayer remnants and small particles (R2) or dead cells (R3). Region R2 is determined based on the single population obtained from no-cell controls (entries #5–8), where shear forces have been applied to disintegrate the multilayered surface. Dot plot similarity between ARS samples (entry #1–2) and PS (entry #9) and PS + ARS (entry #10) controls indicate that no multilayer components were included in the sample suspensions.

Histograms were plotted to correlate number of events gated on live cells (G1–R1) with the respective fluorescence intensity (Fig. 7b). A distinct difference can be observed between cells cultured on PS and PS + ARS where a right shift in cell population is observed for PS + ARS as compared to PS. The shift indicates a slight increase in fluorescence intensity, which is induced by the presence of free unbound ARS. Taking this phenomenon into account, the latter systems were deemed as a more suitable negative control to set the M1 marker to isolate positive (BA-ARS fluorescent) cells from negative populations. Finally, the same marker is applied for the statistical analysis to determine the degree of cellular uptake within positive samples gated for live cells (G1).

**Fig. 8:**
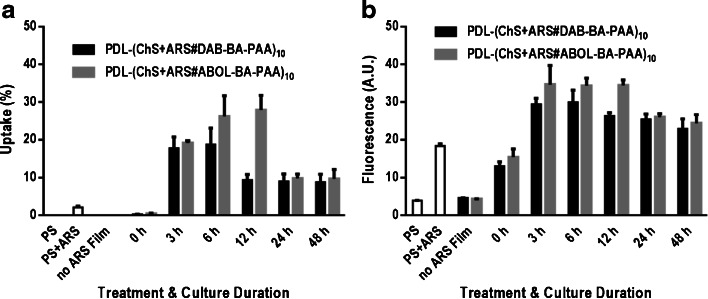
(**a**) Degree of cellular uptake of multilayer components and (**b**) mean fluorescence intensity/cell against cell culture duration.

Figure 8a shows the degree of cellular uptake against different durations of incubation. PS (entry #9), PS + ARS (entry #10), and no-ARS (entry #3–4) control data were collected after 12 h incubation and showed no significant degree of uptake, despite the long culture duration (Fig. 8a, first three bars). The 0 h incubation data was obtained by collecting the FACS sample directly after cell seeding on multilayered surfaces. As the duration of incubation was simply too short for intracellular uptake, any positive signals of positive cells would indicate physical association of burst-released multilayer components on the cell surface. Figure 8a shows that at 0 h, no significant uptake or physical association was detected. Upon longer culture durations, the degree of uptake increased and reached optimal values at 20 and 30% of BA-ARS positive cells for DAB-BA-PAA and ABOL-BA-PAA, respectively, before decreasing to a stable value of 10% after 48 h culture time. Longer culture duration was not attempted as the cells would have grown past confluency, while passage of the confluent cell monolayer to the same surface did not result in substantial degrees of uptake after another 48 h of culture (data not shown). This observation may indicate that the initial burst release observed in Fig. 2 is critical in delivering particles to be taken up by the cells. Following the initial 6 h (for DAB-BA-PAA system) and 12 h (for ABOL-BA-PAA system) incubation, cells proliferated and thus decreased the overall degree of cellular uptake.

Another way to follow the extent of BA-ARS uptake by the cells is by determination of the overall fluorescence intensity in the sample suspension, i.e. not only the cells within the M1 marker, but the whole sample suspension, including the positive values from free ARS without BA (see Fig. 7b). Figure 8b shows that at the respective optimal culture duration, the detected fluorescence intensity was approximately two times higher than that of the 0 h value. Further, as opposed to Fig. 8a where the degree of uptake decreased at least twofold after the optimal culture duration, Fig. 8b shows relatively stable fluorescence intensity indicating that the absolute amount of positive live cells were likely maintained throughout the 48 h culture duration. Fluorescence intensity detected at 0 h was most likely due to burst-released free ARS from the films as supported by a similar fluorescence intensity detected in the 12 h incubated PS + ARS controls (entry #9, Fig. 7b). When the fluorescence intensity of each sample is corrected for the fluorescence intensity of the PS + ARS controls, the resulting fluorescence intensity can be regarded to emerge from ARS-boronate ester formation (see Scheme 2). In that respect, when no-cell controls were subjected to shear forces (entry #5 and 6) in order to measure the relative fluorescence intensity of the multilayer alone, the fluorescence intensity detected was approximately 10-fold the optimal values shown in Fig. 8b. According to the detected fluorescence intensities, the ABOL-BA-PAA system facilitates deposition of ARS and correspondingly more ester formation with the BA-functionality, as compared to the DAB-BA-PAA system.

Noticeable differences between the two BA-PAAs are observed in Fig. 8a where the ABOL-BA-PAA system may sustain cellular uptake of proliferating cells for as long as 12 h, while the DAB-BA-PAA reached the optimal value already after 6 h culture duration. This may be attributed to three factors, i.e. the higher initial incorporation of ARS, enhanced ARS-boronate ester formation, and/or more efficient release of ARS-boronate ester in the former system. As observed in Fig. 2, ABOL-BA-PAA incorporates and releases more ARS during the initial 12 h release phase. Moreover, as shown in Fig. 6 and Scheme 3, ABOL-BA-PAA potentially may also form stronger ARS-boronate esters through the presence of primary alcohols in the vicinity. Next, it was indicated previously that ABOL-BA-PAA polymer self-assembles into monodispersed nano-sized particles that may be internalized more efficiently by cells (15), whereas DAB-BA-PAA provides more disperse particles of lower positive surface charges. As such, albeit showing relatively similar behavior to DAB-BA-PAA throughout the *in vitro* release studies, the ABOL-BA-PAA may eventually provide release of more nanoparticles that are better internalized by cells and contain more stable ARS-boronate ester.

**Fig. 9:**
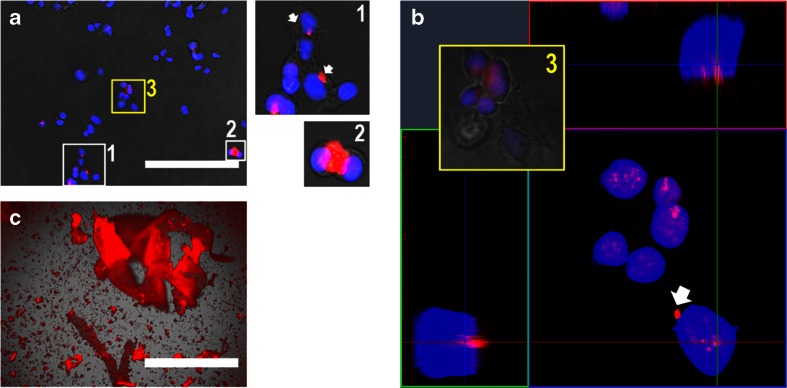
(**a**) Overlaid fluorescence (20X magnification, bar = 200 μm) and (**b**) confocal (40X magnification) microscopy images of COS-7 cells after 20 h culture duration on a (ChS + ARS#ABOL-BA-PAA)_10_ multilayer sample and reseeded onto clean imaging chambers. Insets show higher magnification of selected clusters of cells (*white squares*) and those specifically imaged through confocal microscopy (*yellow square*). (**c**) Overlaid fluorescence image of mechanically disrupted (ChS + ARS#ABOL-BA-PAA)_10_ sample without cells at 4X magnification (bar = 1000 μm).

To assess whether the fluorescent ARS-boronate esters detected by FACS are indeed internalized by cells and not merely physically attached onto the outer membrane of the cells, cells which have been cultured for 20 h were trypsinized, reseeded on imaging chambers, and left to attach for 6 h before staining and fixating for confocal microscopy imaging. Complementing the confocal microscopy, regular fluorescence microscopy was employed to obtain a lower magnification, more global view of the samples. No significant differences were observed between the DAB-BA-PAA and ABOL-BA-PAA systems and therefore images of the latter are shown here as representative results (Fig. 9).

Figure 9b indicates that most of the observed fluorescent particles are indeed taken up by the cells and are not merely adhering to the outer cell membranes. Moreover, these particles co-localize with the Hoechst-stained nuclei. Larger fluorescent aggregates are observed as well, which were physically associated with the cells and/or located at the peri-nuclear region (indicated by arrow). As a control, PDL-(ChS + ARS#ABOL-BA-PAA)_**10**_ was mechanically destroyed and observed under the fluorescence microscope (Fig. 9c), revealing multilayer fragments. These remnants fluoresced brightly under the same conditions indicating that the observed fluorescent particles indeed orifinate from the multilayered surface onto which cells were cultured. No such fluorescence was observed for cells cultured on PS surfaces (data not shown).

## Conclusions

Multilayered thin films from boronic acid-functionalized poly(amido amine)s (BA-PAA) and chondroitin sulfate (ChS) have been evaluated for their abilities in facilitating cellular uptake of the dye alizarin red S (ARS) as a reporter molecule. The dynamic covalent bonding between ARS and BA moieties in the polymers facilitates systematic loading during the multilayer build-up and provides facile characterization through the fluorescence properties of the boronate ester. UV spectroscopy shows linear build-up profiles with distinct λ_max_ indicating successful incorporation of ARS. It is postulated that during build-up, the majority of incorporated ARS was present in dynamic equilibrium with BA moieties of the BA-PAAs, resulting in a stable maximum wavelength of absorption (λ_max_) at 470 nm, corresponding to the λ_max_ of ARS-boronate ester.

The ARS-boronate ester retains pH responsiveness, and more rapid release is obtained upon lowering pH from neutral (pH 7.4) to pH 6, 5, and 4. Concomitant shifts in λ_max_ were observed, in agreement with ARS-boronate ester hydrolysis and ARS’ properties as a pH indicator. Under physiological conditions (pH 7.4), it was proposed that initial burst release of up to 40% (12 h) represents the release of excess ARS, which was initially in dynamic equilibrium within the multilayered films. After 1 h incubation, the λ_max_ shifted very slowly, reaching a plateau at ~510 nm after 20 days of total incubation, indicating the relatively strong ARS-boronate ester formation at pH 7.4. Parallel to the slow increases in λ_max_, ARS was released very slowly, indicating low diffusion coefficients of ARS out of the multilayered structure.

Due to the possibility of competitive boronate ester formation with other diol-containing molecules such as glucose, ARS release was also investigated in the presence of relatively high glucose concentrations. No significant response was observed at glucose concentrations of 25 and 100 mM due to the much higher binding constant of BA to ARS than to glucose. The observation of the λ_max_ change during the glucose incubation experiment indicated that DAB-BA-PAA gave a faster response than ABOL-BA-PAA, i.e. the λ_max_ red-shifted faster. It was hypothesized that primary alcohol side groups in the vicinity of BA reaction center in ABOL-BA-PAA may help by acting as Lewis bases, consequently lowering the pKa of the BA moiety and form stronger boronate esters with ARS. The faster response of DAB-BA-PAA to glucose, however, did not result in enhanced ARS release, which was explained by the slow diffusion of ARS out of the multilayered and rigid film, leaving free unbound ARS trapped inside the film acting as pH indicator (i.e. demonstrating higher λ_max_ at pH 7.4).

For the study of cellular uptake, flow cytometry indicated that compared to the controls, the release of ARS-boronate ester is characterized by significantly higher fluorescence intensity. Cellular uptake was found to be maximal at 20 and 30% following 6 h of culture for DAB-BA-PAA and ABOL-BA-PAA films, respectively. For the latter system, the degree of cellular uptake was maintained for at least another 6 h, most likely through the higher amount and stronger ARS-boronate ester formed with ABOL-BA-PAA. Confocal microscopy further revealed presence of fluorescent particles that were internalized by cells and often co-localized with the cell nuclei, indicating successful delivery of ARS in its boronate ester form with BA-PAAs into the nuclei of the cells cultured on top of the films. Our current research efforts are aimed at exploiting these multilayered thin films as drug delivery systems.

## Abbreviations

ABOL: 4-aminobutanol
ABOL-BA-PAA: p(CBA-ABOL50%/4AMPBA50%)
ARS: Alizarin red S
BA: Boronic acid
BA-PAA: Phenylboronic acid-functional poly(amido amine)s
CBS: Citrate buffered saline
ChS: Chondroitin sulfate
DAB-BA-PAA: p(CBA-DAB50%/4AMPBA50%)
DMEM: Dulbecco’s Modified Eagle’s medium
FACS: Fluorescence-activated cell sorting
LbL: Layer-by-layer
LFH: Laminar flow hood
PDL-TCPS: Poly-D-lysine coated tissue culture polystyrene
PS: Polystyrene
PVA: Poly(vinyl alcohol)
